# Asiatic Acid Interferes with Invasion and Proliferation of Breast Cancer Cells by Inhibiting WAVE3 Activation through PI3K/AKT Signaling Pathway

**DOI:** 10.1155/2020/1874387

**Published:** 2020-02-10

**Authors:** Xiao-jun Gou, Huan-huan Bai, Li-wei Liu, Hong-yu Chen, Qi Shi, Li-sheng Chang, Ming-ming Ding, Qin Shi, Mei-xiang Zhou, Wen-li Chen, Li-min Zhang

**Affiliations:** ^1^Baoshan District Hospital of Integrated Traditional Chinese and Western Medicine of Shanghai, Shanghai 201999, China; ^2^School of Pharmacy, Shaanxi University of Traditional Chinese Medicine, Xianyang, Shaanxi 712046, China; ^3^School of Basic Sciences of Shanxi University of Chinese Medicine, Taiyuan, China

## Abstract

**Objective:**

To explore the ability of asiatic acid to interfere with the invasion and proliferation of breast cancer cells by inhibiting WAVE3 expression and activation through the PI3K/AKT signaling pathway.

**Methods:**

The MDA-MB-231 cells with strong invasiveness were screened by transwell assay, and plasmids with high expression of WAVE3 were constructed for transfection. The transfection effect and protein expression level of plasmids were verified by PCR and WB. The effects of asiatic acid on cell proliferation and invasion were investigated by flow cytometry. The xenografted tumor models in nude mice were established to study the antitumor activity of asiatic acid.

**Results:**

Asiatic acid significantly inhibited the activity of MDA-MB-231 cells, and the expression level of WAVE3 increased significantly in the tissue of ductal carcinoma in situ and was lower than that in the metastasis group. After plasmid transfection, the mRNA and protein expression of WAVE3 increased significantly in the cells. Asiatic acid at different concentrations had an impact on cell apoptosis and invasion and could significantly inhibit the expression of WAVE3, P53, p-PI3K, p-AKT, and other proteins. The T/C(%) of asiatic acid (50 mg/kg) for MDA-MB-231(F10) xenografted tumor in nude mice was 46.33%, with a tumor inhibition rate of 59.55%. Asiatic acid could significantly inhibit the growth of MDA-MB-231 (F10) xenografted tumors in nude mice (*p* < 0.05).

**Conclusions:**

Asiatic acid interferes with the ability of breast cancer cells to invade and proliferate by inhibiting WAVE3 expression and activation and the mechanism of action may be related to the PI3K/AKT signaling pathway.

## 1. Introduction

Breast cancer is one of the most commonly seen malignant tumors in women, which jeopardizes women's physical and mental health and even endangers their lives [1]. In recent years, the incidence of breast cancer has been on the rise in many cities in China and abroad and is on the top of the list of malignancy incidence in women. Moreover, breast cancer tends to occur at a younger age. Considering the medical development at present, it is difficult to accurately explain the pathogenesis of breast cancer, and the occurrence, development, and outcome of the cancer are yet to be fully understood. The current approach to treat breast cancer is mainly surgery, combined with postoperative radiotherapy, chemotherapy, endocrine therapy, and so forth. Although the treatment has been relatively perfect, there are still many patients experiencing recurrence and metastasis, and the side effects of radiotherapy and chemotherapy and drug resistance have also become obstacles in the treatment of many patients [2]. Therefore, more and more attention has been paid to the role of traditional Chinese medicine in the treatment of breast cancer.

Tumor metastasis is a major challenge in the treatment of cancer. Metastasis accounts for more than 90% of cancer-related deaths since it is difficult to treat by surgery or conventional chemotherapy and radiotherapy. Therefore, it is important to search for drugs preventing tumor metastasis [3]. Asiatic acid affects migration, invasion, and apoptosis of colon cancer SW480 and HCT116 cells; it regulates Pdcd4 through the PI3K/Akt/mTOR/p70S6K signaling pathway and inhibits migration and invasion and induces apoptosis of the colon cancer cells [4, 5]. Abnormality of signal transduction pathway is an important step in the occurrence and development of tumor, and the PI3K/Akt/mTOR signaling pathway is closely related to a variety of human tumors, playing an important role in the proliferation, survival, resistance to apoptosis, angiogenesis and metastasis of tumor cells, and resistance to radiotherapy and chemotherapy of the cells. Abnormal activation of the PI3K/Akt/mTOR pathway is seen frequently in breast cancer, and drugs targeting this pathway have been a research focus in the treatment of cancer [6].

Asiatic acid (AA), extracted from the root of a Chinese herbal medicine, *Actinidia valvata Dunn* (Actinidiaceae), is a pentacyclic triterpenoid acid plant polyphenols [7]. It has been shown in studies [8–10] that asiatic acid reduces inflammation and depression, cares for the skin, benefits the liver and the lungs, lowers blood sugar and lipid, and induces apoptosis of tumor cells. Its antitumor effects are mainly seen in the induction of cell death through a mitochondrial-mediated pathway [11].

As an important intracellular signal transduction pathway, the PI3K-AKT signaling pathway plays an important role in cell apoptosis and survival [12]. This pathway is necessary for the regulation of cell proliferation, differentiation, and apoptosis, is an important way to promote cell survival and proliferation, prevents cells from apoptosis, and assists angiogenesis and tolerance to chemotherapy. PI3K phosphatidylinositol is a component of the eukaryotic cell membrane, the head of the phosphatidylinositol can be phosphorylated by phosphoinositide kinase (PI3K), and PI3K, as a signal transducer, participates in the regulation of various cellular functions [13].

Phosphatidylinositol 3-kinase (PI3K) is a phosphatidylinositol kinase that phosphorylates the third hydroxyl of the inositol ring. The activation of PI3K results in the production of a second messenger, PIP3, on the plasma membrane. PIP3 binds to AKT, a signal protein with PH domain, and to phosphoinositide-dependent kinase-1 (PDK1), promoting PDK1 to phosphorylate thr308 of the AKT protein, which can be fully activated through the phosphorylation of Ser473 by PDK2, e.g., integrin-linked kinase (ILK) [14, 15].

AKT is a serine/threonine kinase, which consists of 480 amino acid residues. It is highly homologous with protein kinase A (PKC) and protein kinase C (PKC) and is one of the main downstream effectors of PIK3. The activated AKT is transferred from cell membrane to cytoplasm and nucleus, where it activates or inhibits the downstream target proteins including Bad, Caspase-9, Tuberin, GSK3b, Forkhead, and mTOR by phosphorylation and regulates cell proliferation, apoptosis, and migration [16, 17].

Therefore, PI3K and AKT may be potential targets for tumor treatment.

WAVE3, a member of the Wiskott-Aldrich syndrome protein (WASP) family, is a regulatory protein for actin and is highly expressed in malignant breast cancer. It has a profound impact on the movement and invasion of breast cancer cells, and its absence or abnormal expression causes cells to show abnormality in cell membrane structure, actin polymerization, cell migration and invasion, and so forth. [18]. WAVE3-mediated pseudopodia formation and cell migration require the presence of the product phosphatidylinositol 3,4,5-triphosphate (PIP3), which is activated by PI3K. At the same time, the regulatory subunit p85 of PI3K can also bind to the phosphorylated WAVE3 to promote the migration of pseudopodocytes [19]. In the investigation of the regulatory mechanism of WAVE3, it has been revealed that the regulatory subunit p85 of PI3K mediates the action of WAVE3 through binding of the SH2 domain at its C-terminal to WAVE3. The expression of WAVE3 in human breast adenocarcinoma MDA-MB-231 cells has been inhibited, leading to a significant reduction in the movement, migration, and invasion of the cells, and migration and invasion are crucial to the ability of tumor cells to metastasize locally and remotely [20].

## 2. Materials and Methods

### 2.1. Experimental Materials

#### 2.1.1. Cell Lines

The human breast cancer cell lines MCF-7 and MDA-MB-231 were cultured in DMEM containing 10% fetal bovine serum (FBS).

#### 2.1.2. Reagents and Consumables

DMEM (high sugar) was obtained from Invitrogen Gibco (Grand Island, NY, USA), FBS from Science Cell Research Laboratories (Carlsbad, CA, USA), and CCK8 cell viability test kit from Nanjing Enogene Biotech Co., Ltd. (Nanjing, China). Asiatic acid was supplied by the client. Transwell chambers with a pore size of 8.0 *μ*m were purchased from Corning Incorporated (Corning, NY, USA), with REF 3422 and LOT 14416045, and BD Matrigel Matrix (Basement Membrane) from BD Biosciences (San Jose, CA, USA), and anti-WAVE3 rabbit anti-human/mouse antibody E 20–74899 from Nanjing Enogene Biotech. Anti-P53 rabbit anti-human/mouse antibody, E11-10276C; EnoGene anti-NF-*κ*B rabbit anti-human antibody, E10-20406; EnoGene anti-p-PI3K rabbit anti-human/mouse antibody, E011508-2 and E1A7005A; and EnoGene anti-GAPDHrabbit anti-human/mouse antibody, E90062, were obtained from Nanjing Enogene Biotech Co., Ltd. (Nanjing, China). The hydrophobic PVDF membrane was purchased from Merck Millipore (Billerica, MA, USA). ECL chemiluminescence reagent was obtained from Beyotime Biotechnology (Shanghai, China), and P0018A, RIPA lysate, BSA, prestaining protein marker, HRP labeled goat anti-rabbit secondary antibody, developer, fixing solution, and BCA protein concentration test kit were purchased from Nanjing Enogene Biotech. Co., Ltd (Nanjing, China).

#### 2.1.3. Instruments

The uses of the instruments were shown in Table 1.

### 2.2. Experimental Methods

#### 2.2.1. Determination of the Proliferation Activity of Asiatic Acid on Human Cancer Cell Lines *In Vitro* by CCK-8 Cell Proliferation Activity Kit

Tumor cells were cultured separately, and cells in the logarithmic growth phase were inoculated into a 96-well plate at 1 × 10^5^ cells/mL, 100 *μ*L/well, at 37°C, 5% CO_2_ for 24 h. Asiatic acid at corresponding concentrations was added separately and there was a negative control group too. After incubation with cells for 72 h, the growth status of cells in each group was observed under a microscope. 10 *μ*L CCK8 was added to each well and left at room temperature for 4 h. Absorbance was detected at 450 nm, and IC_50_ was calculated.

#### 2.2.2. Breast Cancer Cells with Strong Invasive Ability Were Screened Out by Transwell Assay for Follow-Up Study, 10 Passages in Total: The MDA-MB-231 Cells Obtained Were Named MDA-MB-231 (F10)

The transwell chambers coated with matrix glue were put into a culture plate, and 300 *μ*L serum-free medium was added in the upper chamber and left at room temperature for 15–30 min to rehydrate the matrix glue. Then, the remaining culture medium was removed. The cells were starved for 12 h and then resuspended in serum-free medium containing BSA to prepare cell suspension, and the density was adjusted to 1 × 105 cells/mL. 500 *μ*L of the cell suspension was inoculated into the transwell chambers, 500 *μ*L of medium containing FBS was added to the lower chamber for routine culture for 24 h, and the cells in the lower chamber were collected for further culture. When the cells in the lower chamber proliferate to a certain number, the transwell chambers coated with matrix glue continued to be used to screen out the cells invading the lower chamber, and the process was repeated for 10 passages to finally obtain the MDA-MB-231 cells with high invasive ability, which were named MDA-MB-231 (F10). The matrix glue and the cells in the upper chamber were wiped off with cotton swabs and stained with 0.1% crystal violet. The number of penetrating cells was counted under a microscope. The cells were decolorized with 33% acetic acid to completely elute the crystal violet and the eluent was collected. OD value was detected at 570 nm to indirectly show the number of cells.

#### 2.2.3. WAVE3 Expression Was Determined in Tumor Tissue and Normal Breast Tissue of Patients with Breast Cancer by Immunohistochemistry

Tumor tissue and normal breast tissue of the patients were collected to make paraffin sections. The paraffin sections were placed in an oven at 67°C for 2 h, dewaxed and hydrated, and rinsed three times with PBS at pH7.4, 3 min each time. A certain amount of citrate buffer at pH = 6.0 was added to a microwave box and heated to boiling by microwave. The dewaxed and hydrated tissue sections were placed on a high-temperature-resistant plastic section rack, which was put into the boiling buffer and microwaved with midrange power for 10 min. The microwave box was removed and cooled naturally with running water. The slides were taken out of the buffer, rinsed twice with distilled water, and then rinsed twice with PBS, 3 min each time. Each section was added with one drop of 3% H_2_O_2_, incubated at room temperature for 10 min to block the activity of endogenous peroxides, and rinsed three times with PBS, 3 min each time. The PBS solution was removed. Each section was added with one drop of corresponding primary antibodies (using corresponding dilution factor) and incubated at room temperature for 2 h. The sections were rinsed with PBS three times, 5 min each time. The PBS solution was removed. Each section was added with one drop of polymer enhancer and incubated at room temperature for 20 min. The sections were rinsed with PBS three times, 3 min each time. The PBS solution was removed. Each section was added with one drop of enzyme-labeled anti-mouse/rabbit polymer and incubated at room temperature for 30 min. The sections were rinsed with PBS three times, 5 min each time. The PBS solution was removed. Each section was added with one drop of freshly prepared DAB solution (diaminobenzidine) and observed under a microscope. The sections were restained with hematoxylin, differentiated with 0.1% HCl, and rinsed with tap water to show the color of blue. They were dehydrated and dried with gradient alcohol. Xylene was used for the sections to be transparent. Then, the sections were sealed with neutral gum and observed after drying.

#### 2.2.4. The Human Breast Cancer Cell Lines MDA-MB-231 and the Expression of WAVE3 Gene and Protein after Transfection of MDA-MB-231 (F10) Were Studied by Real-Time PCR and Western Blot

*(1). Extraction of Total RNA*. The cells were lysed in TRIpure Regent, 1 mL for each well in a 6-well plate. Total RNA precipitate was obtained after the lysate was subject to extraction with chloroform, precipitation with isopropanol, and washing with 75% ethanol.

*(2). Reverse Transcription*. Total RNA was reverse-transcripted into cDNA following the instructions of the corresponding reverse transcription kits, and the expression of each target gene was detected by real-time quantitative PCR, using GAPDH as an internal reference.

The specific reaction conditions were as follows.

When the RNA primer mixture (Table 2) was prepared in a PCR tube, the reaction was carried out at 65°C for 10 min, and then the mixture was quickly cooled on ice for at least 2 min.

The reverse transcription reaction solution (Table 3) was added to the above PCR tube to a total volume of 20 *μ*l. After centrifugation for a few seconds, the reaction was started on a PCR instrument at 42°C for 50 min and 85°C for 5 min. When the reaction was terminated, cDNA was cooled on ice and stored at 4°C for PCR amplification.

*SYBR Green Real-Time PCR*. The PCR reaction solution was prepared with the components mentioned in Table 4. Centrifugation was performed briefly to ensure that all reaction fluids were at the bottom of the well. The reaction was performed in triplicate for each sample and each gene. PCR amplification conditions are shown in Table 5.

*Analysis of Melting Curve*. The analysis was performed at 60°C to 95°C with an interval of 0.5°C, the reaction lasted for 5 sec each, and step detection was adopted. Data were calculated following the above experimental method and thresholds and Ct values were automatically obtained by the software.

#### 2.2.5. Western Blot

The first step is cell lysis, where the cells were washed once with 1×PBS, added with RIPA 300 *μ*L/well, and shaken at 4°C for 30 min. Then, the cells in each well were repeatedly aspirated with a pipette and centrifuged at 4°C, 10000 rpm for 10 min. The cell lysate was transferred to a 1.5 mL centrifuge tube to obtain the cell protein sample, which was stored at −80°C. Second, the SDS denatured 10% polyacrylamide gel was prepared at the ratio in Table 6 (lower layer separation gel, single side). After mixing, the gel was quickly filled to 2/3 of the total height of the glass plate, and then 1 mL of water-saturated n-butyl alcohol was added above the gel to ensure that the upper layer of the gel was smooth. The gel stood for it to set.

The SDS denatured 5% polyacrylamide gel was prepared at the ratio in Table 7 (upper layer stacking gel, single side). After mixing, the gel was quickly filled to the total height of the glass plate and the comb was inserted. The gel stood for it to set. Before electrophoresis, the comb was removed, the gel was placed in the 1 × Tris-glycine electrophoresis buffer, and the loading wells were blown clean with a syringe needle.

The protein sample was mixed with 5 × loading buffer (containing *β*-mercaptoethanol), denatured by boiling for 5 min, and placed in an ice bath for 5 min. An appropriate amount of protein samples was loaded, and SDS denatured 10% polyacrylamide gel electrophoresis (SDS-PAGE) was performed until the target protein was separated effectively. After electrophoresis, the gel was removed and placed in a sandwich holder designed for transfer film, with the gel in the negative electrode and the nitrocellulose membrane in the positive electrode. Transfer film was performed in transfer film buffer at 4°C, 300 mA constant current for 2 h so that proteins in the gel were transferred to the nitrocellulose membrane and form blots. The membrane was put in 1 × Blotto, closed, and shaken at room temperature for 1 h. The membrane was cut at the blots of the detected protein molecular weight, placed in Blotto containing corresponding primary antibodies, and shaken overnight at 4°C. The membrane was placed in 1 × TBST solution and rinsed by shaking for 5 min, 4 times in total. The membrane was placed in a developer (Western Lightning™ Chemiluminescence Reagent) for 1 min. The membrane was placed immediately in the exposure box. The photographic film was exposed for 1 min in the darkroom and then developed and fixed.

#### 2.2.6. Transwell Assay to Detect Changes in Cell Invasion

The first step is transwell assay, where the transwell chambers coated with matrix glue were put into a culture plate, and 300 *μ*L preheated serum-free medium was added in the upper chamber and left at room temperature for 15–30 min to rehydrate the matrix glue. Then, the remaining culture medium was removed. The cells were starved for 12 h and then resuspended in serum-free medium containing BSA to prepare cell suspension, and the density was adjusted to 1 × 10^5^ cells/mL. 100 *μ*L of the cell suspension was inoculated into the transwell chambers, and 500 *μ*L of medium containing FBS was added to the lower chamber for routine culture for 12∼48 h. The matrix glue and the cells in the upper chamber were wiped off with cotton swabs and stained with 0.1% crystal violet. The number of penetrating cells was counted under a microscope. The cells were decolorized with 33% acetic acid to completely elute the crystal violet and the eluent was collected. OD value was detected at 570 nm to indirectly show the number of cells.

#### 2.2.7. The Expression of WAVE3, P53, NF-KB(P65), p-PI3K, t-PI3K, p-AKT, and t-AKT Was Detected by WB after the MDA-MB-231 (F10) Cells Were Treated with Asiatic Acid at Different Concentrations for 72 Hours

This step uses WB technology.

#### 2.2.8. The Tumor Effect and Activity Intensity of Asiatic Acid on MDA-MB-231 (F10) Xenograft in Nude Mice Were Studied

The MDA-MB-231 (F10) cells in the logarithmic growth phase were inoculated subcutaneously in the right armpit of 30 nude mice under sterile conditions, 5 × 10^6^ cells per mouse. The diameter of the tumor was measured with a vernier caliper. When the tumor grew to about 80 mm^3^, 24 nude mice in good condition which had tumor size in good uniformity were selected and randomly divided into four groups, six animals in each group, i.e., model group, low-dose group, high-dose group, and positive drug group. Asiatic acid was given by gavage for 14 days, 200 *μ*L/animal. The model group was given vehicle control of the same volume. The tumor inhibitory effect of the test substance was observed dynamically by measuring the tumor diameter. The tumor volume was measured every three days, and the weight of the mice was measured at the same time.

#### 2.2.9. Statistical Analysis

Data were expressed in mean ± SD and analyzed with the statistical software GraphPad Prism 5.0. *T*-test was used for comparison between the two groups, and one-way ANOVA (Dunnett) was used for the comparison between multiple groups. *p* < 0.05 indicated statistical significance.

## 3. Results

### 3.1. Effect of Asiatic Acid on Proliferation of the Tumor Cells MDA-MB-231 and MCF-7 *In Vitro*

**Figure 1:**
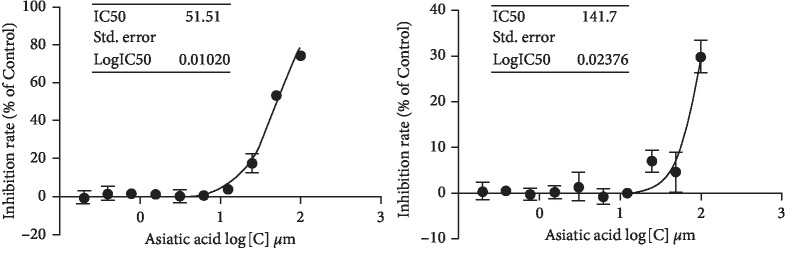
Effect of asiatic acid on the proliferation of human breast cancer cell lines (a) MCF-7 and (b) MDA-MB-231 *in vitro.*^*∗*^(*.lpl*) < 0.05 (mean ± SD, *n* = 5).

As shown in Figure 1, asiatic acid could significantly inhibit the proliferation of the tumor cells MDA-MB-231 and MCF-7 *in vitro*, with the strongest inhibitory activity on MDA-MB-231(*p* < 0.05).

### 3.2. Screening of the Cell Line MDA-MB-231 with High Invasiveness

**Figure 2:**
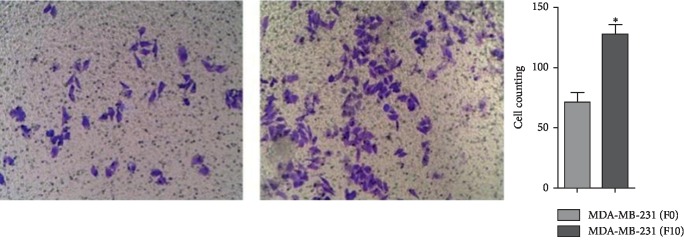
Difference of invasiveness between human breast cancer cells MDA-MB-231 (F0) and MDA-MB-231 (F10) *in vitro.*^*∗*^*p* < 0.05 (mean ± SD, *n* = 5).

**Figure 3:**
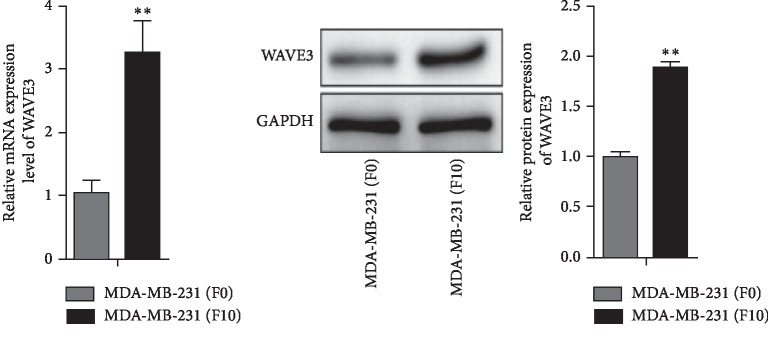
Difference in expression of WAVE3 protein between human breast cancer cells MDA-MB-231 (F0) and MDA-MB-231 (F10). ^*∗*^*p* < 0.05 (mean ± SD, *n* = 3).

The breast cancer cells MDA-MB-231 were quite invasive (*p* < 0.05). The MDA-MB-231 (F0) cells were more invasive than the MDA-MB-231 (F10) cells (*p* < 0.05) (Figure 2). And the expression of WAVE3 protein and gene was higher in MDA-MB-231 (F10) than in MDA-MB-231 (F0) (*p* < 0.05) (Figure 3).

### 3.3. Effects of WAVE3 Knockout and High Expression on Proliferation and Invasion of MDA-MB-231 (F0) and MDA-MB-231 (F10) and Intervention of Asiatic Acid

**Figure 4:**
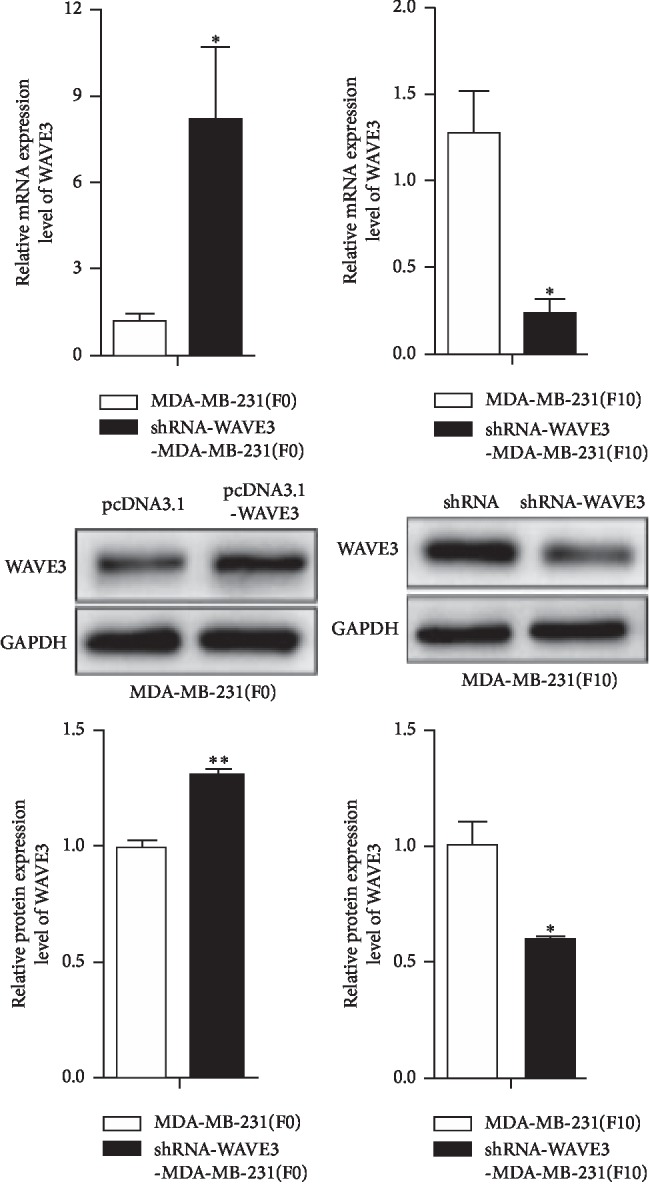
Expression of WAVE3 gene and protein before and after transfection of MDA-MB-231 (F0) and MDA-MB-231 (F10). ^*∗*^*p* < 0.05 (mean ± SD, *n* = 2).

As shown in Figure 4, when the MDA-MB-231 (F0) cells were transfected with the pcDNA3.1-WAVE3 plasmid, the expression of WAVE3 mRNA and protein increased significantly (*p* < 0.05). The expression of WAVE3 mRNA and protein decreased significantly in the MDA-MB-231 (F10) cells after knockout of WAVE3 (*p* < 0.05).

### 3.4. Role of WAVE3 in Metastasis of Breast Cancer

**Figure 5:**
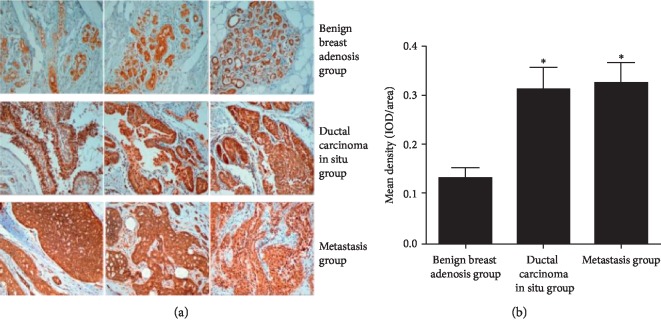
Expression of WAVE3 in tissues detected by immunohistochemistry in benign breast adenosis group, ductal carcinoma in situ group, and metastasis group. ^*∗*^*p* < 0.05 (mean ± SD, *n* = 20).

As shown in Figure 5, the grouping results of different pathological sections showed that the benign breast adenosis group was confirmed to be patients with fibroadenoma of breast, the ductal carcinoma in situ group was confirmed to be patients with ductal carcinoma in situ, and the metastasis group was confirmed to be breast cancer patients with lymph node metastasis. The expression of WAVE3 increased significantly in ductal carcinoma in situ tissue, and it was even higher in the metastasis group (*p* < 0.05). WAVE3 might be involved in drug resistance, invasion, and metastasis of tumor cells.

### 3.5. Effects of Asiatic Acid on Apoptosis and Invasion of MDA-MB-231 (F0) and MDA-MB-231 (F10) before and after Transfection with WAVE3

**Figure 6:**
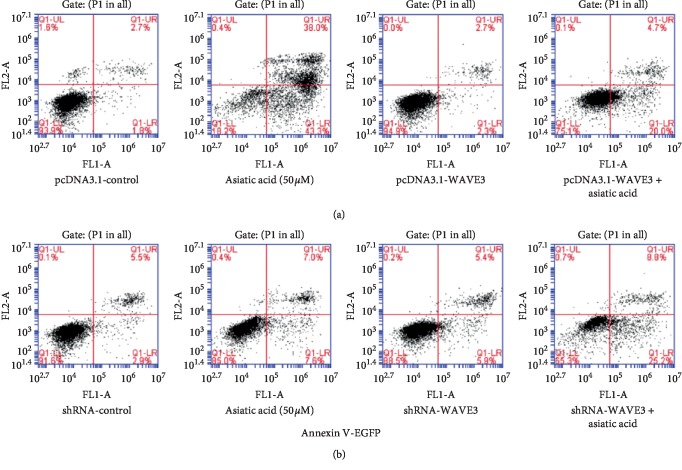
Effects of asiatic acid on apoptosis of (a) MDA-MB-231 (F0) and (b) MDA-MB-231 (F10) before and after transfection with WAVE3.

**Figure 7:**
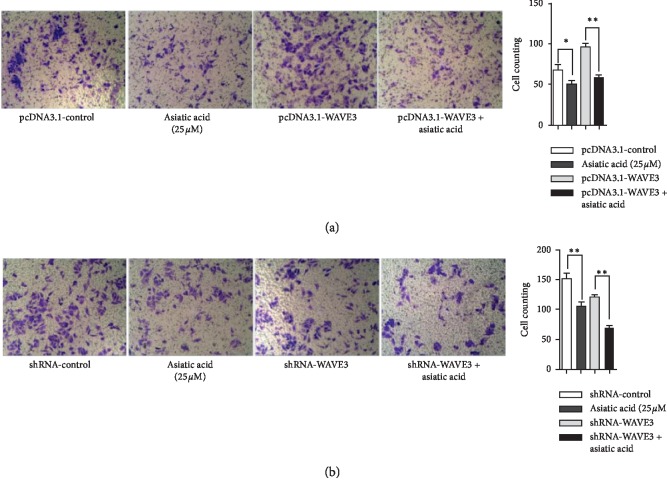
Effects of asiatic acid on the invasiveness of (a) MDA-MB-231 (F0) and (b) MDA-MB-231 (F10). ^*∗*^*p* < 0.05, ^*∗∗*^*p* < 0.01.

Asiatic acid could reduce the invasiveness of MDA-MB-231 (F0) and MDA-MB-231 (F10) and also had an impact on the invasiveness of the cells by interfering with the expression of WAVE3 (Figure 6). Asiatic acid (50 *μ*M) could significantly induce apoptosis of MDA-MB-231 (F0). After transfection with pcDNA3.1-WAVE3, the ability of asiatic acid to induce apoptosis was weakened (*p* < 0.01). Asiatic acid (50 *μ*M) had a weaker ability to induce apoptosis for MDA-MB-231 (F10) than MDA-MB-231 (F0), but after transfection with shRNA-WAVE3, the ability of asiatic acid to induce apoptosis of MDA-MB-231 (F0) was improved (*p* < 0.01). Asiatic acid (25 *μ*M) could reduce the invasiveness of MDA-MB-231 (F0) and MDA-MB-231 (F10) (*p* < 0.05, *p* < 0.01) and also had an impact on the invasiveness of the cells by interfering with the expression of WAVE3 (Figure 7).

### 3.6. After Treatment of the MDA-MB-231 (F10) Cells with Asiatic Acid at Different Concentrations for 72 h

**Figure 8:**
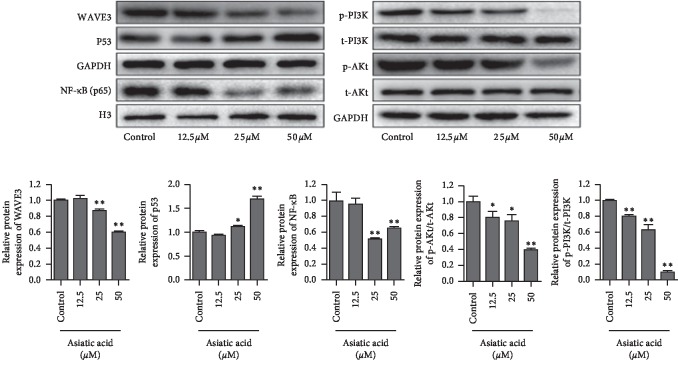
Expression of WAVE3, P53, NF-KB (P65), p-PI3K, t-PI3K, p-AKT, and t-AKT after the treatment of the MDA-MB-231 (F10) cells with asiatic acid at different concentrations for 72 h. ^*∗*^*p* < 0.05, ^*∗∗*^*p* < 0.01.

As shown in Figure 8, the cells were collected, the proteins were extracted, and the expression of WAVE3, P53, NF-KB(P65), p-PI3K, t-PI3K, p-AKT, and t-AKT was detected by WB. Asiatic acid could significantly inhibit the expression of WAVE3, P53, NF-KB(P65), p-PI3K, and p-AKT, but it had no significant effect on the expression of t-PI3K and t-AKT (*p* < 0.05, *p* < 0.01).

### 3.7. Evaluation of Tumor Inhibitory Effect and Activity Intensity of Asiatic Acid on MDA-MB-231 (F10) Xenograft in Nude Mice

**Figure 9:**
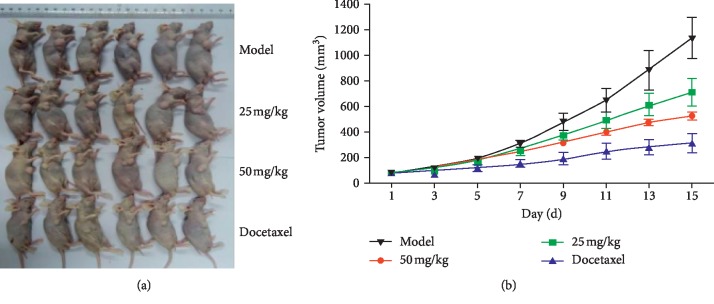
Effects of asiatic acid on changes in growth volume of the drug-resistant human breast cancer cell line MDA-MB-231 (F10) xenografted tumor in nude mice.

**Figure 10:**
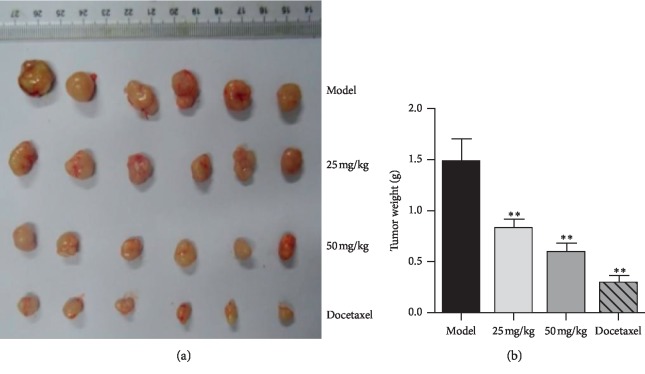
Effects of asiatic acid on the weight of the drug-resistant human breast cancer cell line MDA-MB-231 (F10) xenografted tumor in nude mice. Compared with the model group, ^*∗*^*p* < 0.05, ^*∗∗*^*p* < 0.01.

**Figure 11:**
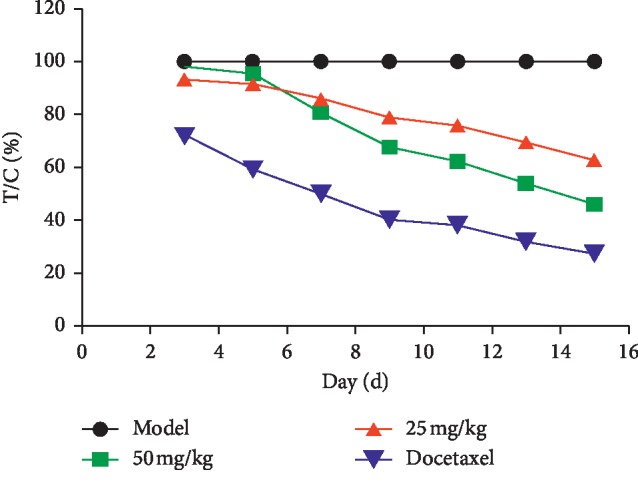
Effects of asiatic acid on tumor inhibitory rate of the drug-resistant human breast cancer cell line MDA-MB-231 (F10) xenografted tumor in nude mice. Compared with the model group, ^*∗*^*p* < 0.05, ^*∗∗*^*p* < 0.01.

**Figure 12:**
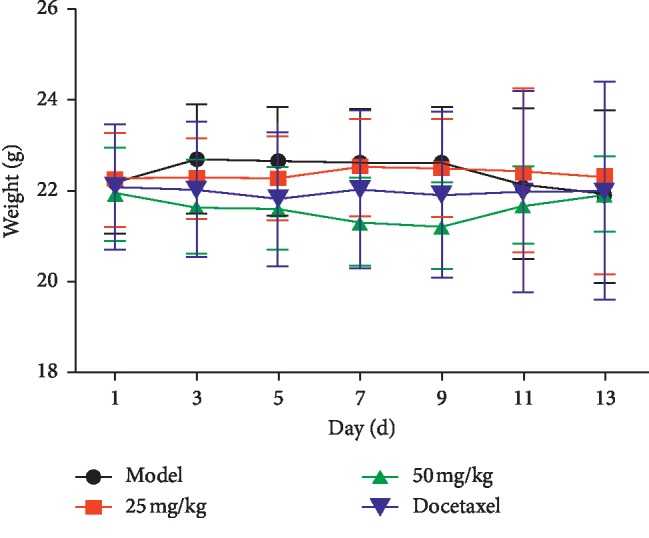
Effects of asiatic acid on the weight of the nude mice with drug-resistant human breast cancer cell line MDA-MB-231 (F10) xenografted tumor. Compared with the model group, ^*∗*^*p* < 0.05,^*∗∗*^*p* < 0.01.

The xenografted tumor model in nude mice was established by using the human breast cancer cell line MDA-MB-231 (F10) with high invasiveness. Asiatic acid at different doses was given by gavage for 14 consecutive days. Tumor volume was measured every two days (Figure 9). After dosing, the tumor was weighed to calculate the tumor inhibitory rate (*p* < 0.05, *p* < 0.01) (Figure 10). Results showed that asiatic acid (50 mg/kg) significantly inhibited the growth of MDA-MB-231 (F10) xenografted tumor in nude mice, with T/C(%) (the percentage of the tumor volume of the drug group divided by the tumor volume of the control group) of 46.33% and a tumor inhibitory rate of 59.55% (*p* < 0.05, *p* < 0.01) (Figures 11 and 12).

## 4. Discussion

It has been suggested in current studies that asiatic acid may become one of the important multitarget drugs from natural sources, which can be used for further drug development and clinical application [21]. In the past few years, asiatic acid has been proved to be a potential anticancer compound, and it has been demonstrated in multiple studies [22–25] that asiatic acid has an inhibitory effect on cancer of the liver, brain, ovary, and lungs. Specifically, asiatic acid has outstanding advantages in reducing inflammation, caring for the skin, and protecting the liver and nerves. It protects the skin from light damage [26, 27], prevents liver fibrosis [28, 29], protects neurons [30, 31],and so forth, and hence it is better at preventing and fighting against skin cancer, liver cancer, glioma, and glioblastoma. Currently, asiatic acid has not been used clinically in the treatment of patients with a tumor. However, the safety and pharmacokinetics of the compound were evaluated in phase I clinical trial on a capsule prepared for asiatic acid (ECA 233) in healthy volunteers. None of the volunteers discontinued due to adverse reactions during the trial, indicating that asiatic acid was safe [32]. Since asiatic acid, an important extract from *Actinidia valvata Dunn*, has an anticancer effect, the inhibitory effect of AA on the proliferation of the breast cancer cells MCF-7 and MDA-MB-231 was investigated first of all in this experiment. Results showed that asiatic acid could significantly inhibit the proliferation of the MDA-MB-231 and MCF-7 cells *in vitro*, with the strongest inhibitory activity on MDA-MB-231. In breast cancer, the MDA-MB-231 cells of 10 passages in total with strong invasiveness were screened out by transwell assay and were named MDA-MB-231 (F10). pcDNA3.1-WAVE3 and shRNA-WAVE3 were established separately, the MDA-MB-231 cells were transfected with pcDNA3.1-WAVE3 to highly express WAVE3, and the MDA-MB-231 (F10) cells were transfected with shRNA-WAVE3 to result in reduced expression of WAVE3. Results showed that the expression of WAVE3 mRNA and protein increased significantly in the MDA-MB-231 (F0) cells transfected with the pcDNA3.1-WAVE3 plasmid, and the expression of WAVE3 mRNA and protein decreased significantly in the MDA-MB-231 (F10) cells with WAVE3 knocked out.

Then, the effect of WAVE3 expression level on the ability of asiatic acid to induce apoptosis and inhibit invasion of tumor cells was investigated. Results showed that asiatic acid (50 *μ*M) could significantly induce apoptosis of the MDA-MB-231 (F0) cells, but this ability was reduced when the cells were transfected with pcDNA3.1-WAVE3. The ability of AA (50 *μ*M) to induce apoptosis was weaker for the MDA-MB-231(F10) cells than for the MDA-MB-231 (F0) cells. After transfection with shRNA-WAVE3, however, the ability of AA to induce apoptosis was improved for the MDA-MB-231 (F0) cells. AA (25 *μ*M) could reduce the invasiveness of MDA-MB-231 (F0) and MDA-MB-231 (F10) and also had an impact on the invasiveness of the cells by interfering with the expression of WAVE3. Moreover, it was investigated whether AA interfered with proliferation and invasion of the breast cancer cells by inhibiting activation of WAVE3 through the PI3K/AKT signaling pathway. It was found that AA could significantly inhibit the expression of WAVE3, P53, NF-KB(P65), p-PI3K, and p-AKT but had no significant effect on the expression of t-PI3K and t-AKT.

Finally, the MDA-MB-231 (F10) xenografted tumor model in nude mice was established to investigate the antitumor activity of AA *in vivo*. The experimental results revealed that the T/C (%) of AA (50 mg/kg) for MDA-MB-231(F10) xenografted tumor in nude mice was 46.33%, with a tumor inhibition rate of 59.55%. AA could significantly inhibit the growth of MDA-MB-231 (F10) xenografted tumors in nude mice (*p* < 0.05).

Asiatic acid can significantly inhibit the proliferation of the MDA-MB-231cells *in vitro*, indicating that it is advisable to treat breast cancer with Chinese herbal medicine, *Actinidia valvata Dunn*. Local invasion and distant metastasis are a challenge in the treatment of breast cancer at present, and our experimental results provide a reliable basis for subsequently studying AA that exerts an antitumor effect by inhibiting WAVE 3. AA at a concentration of 50 *μ*M induces apoptosis of breast cancer cells, while AA at 25 *μ*M reduces the invasive ability of the cells. In the determination of the expression level of WAVE 3 by immunohistochemistry, it was revealed that the expression level of WAVE 3 increased significantly in the tissues of ductal carcinoma in situ and was lower than that in the metastasis group, indicating possible participation of WAVE 3 in the formation of drug resistance, invasion, and metastasis of the tumor cells. It suggests that the proliferation of cancer cells can be inhibited by changing the concentration of asiatic acid and interfering with WAVE3 expression. The success of the *in vitro* model will provide a theoretical basis for clarifying the mechanism of action of AA in inhibiting invasion and proliferation of breast cancer cells and for further conduction of relevant studies.

The tumor occurs mainly due to disorder in the dynamic balance between cell proliferation and apoptosis. The PI3K/AKT signaling pathway is necessary for the regulation of cell proliferation and apoptosis, and its activation is closely related to human breast cancer. The PI3K/AKT signaling pathway can be inhibited by gene knockout or small molecule drugs, which blocks the activation of many downstream antiapoptotic effects for molecules, promotes cell apoptosis, effectively inhibits tumor growth, and increases the sensitivity of cancer cells to radiotherapy and chemotherapy to improve the efficacy [5]. The PI3K/AKT gene promises to be a new target for the treatment of multiple tumors related to hyperactivity of the PI3K-AKT signaling pathway and this also provides a new strategy for clinical application of gene intervention in the treatment of malignant tumors.

## 5. Conclusions

In this study, the inhibitory effect of asiatic acid was investigated on the proliferation of breast cancer cells MCF-7 and MDA-MB-231. The MDA-MB-231 cells with high invasiveness were screened out by transwell assay to be used for subsequent study, 10 passages in total. The MDA-MB-231 cells obtained were named MDA-MB-231 (F10). The pcDNA3.1-WAVE 3 and shRNA-WAVE 3 plasmids were made separately. The MDA-MB-231 cells were transfected with pcDNA3.1-WAVE 3 to express a high level of WAVE 3, and the MDA-MB-231 (F10) cells were transfected with shRNA-WAVE 3 to express a reduced level of WAVE 3. The effect of WAVE 3 expression level was investigated on the ability of AA to induce apoptosis of and inhibit invasion of breast cancer cells, it was studied whether AA interfered with proliferation and invasion of the cells by inhibiting WAVE 3 activation through the PI3K-AKT signaling pathway, and the MDA-MB-231 (F10) xenografted tumor in nude mice was established to investigate the antitumor activity of AA *in vivo*.

In conclusion, it is advisable to treat breast cancer with asiatic acid, an important extract from *Actinidia valvata Dunn*. AA inhibits the expression and activation of WAVE 3 and interferes with the ability of the cancer cells to proliferate and invade. The mechanism of action may be related to signal transduction of the PI3K/AKT signaling pathway.

This will provide a solid theoretical basis for subsequent studies to investigate the inhibitory effect of asiatic acid on breast cancer, the PI3K/AKT signaling pathway, inhibition of WAVE 3 activation by AA, and the effect of AA on proliferation and invasion of breast cancer cells.

## Figures and Tables

**Table 1:** Instruments.

Name of instruments	Manufacturer	Instrument model
Biosafety cabinet	Suzhou Purification Equipment Co., Ltd.	BHC-1300 A/B2
Carbon dioxide incubator	SANYO	MCO-15AC
Inverted fluorescence biomicroscope	Nanjing Jiangnan Novel Optics Co., Ltd.	XD-202
Table-top high speed centrifuge	SCILOGEX	D2012
Low temperature high speed centrifuge	SCILOGEX	D3024 R
Microplate reader	Thermo Scientific	MUTISKAN MK3
Precision electronic balances	Satorius	BSA224S
Minitype vertical electrophoresis tank	Tanon Science & Technology Co., Ltd.	VE 180
Transfer electrophoresis tank	Tanon Science & Technology Co., Ltd.	VE 186
Electrophoresis apparatus	Tanon Science & Technology Co., Ltd.	EPS 300
Decoloring shaker	Jintan Ronghua Instrument Manufacturing Co., Ltd.	TY-80B
Mute mixer	Haimen Kylin-Bell Lab Instruments Co., Ltd.	WH-986

**Table 2:** RNA primer volume.

Components	Volume	Final concentration
Total RNA	2 *μ*g	2 *μ*g/rxn
Oligo dT primer (10 *μ*M)	1 *μ*l	0.5 *μ*M
dNTPs (10 mM each)	1 *μ*l	500 *μ*M
RNase-free water	Up to 14.5 *μ*l	

**Table 3:** Reverse transcription reaction solution.

Reagent components	Volume	Final concentration
Inhibitor (40 U/*μ*l)	(20 U)	20 U/rxn
5X RT buffer	4 *μ*l	1X
EasyScriptTMRTase (200 U/*μ*l)	1 *μ*l (200 U)	20 U/rxn

**Table 4:** PCR reaction solution.

Reagent components	Volume	Final concentration
2X qPCR MasterMix	10 *μ*l	1X
Forward primer (10 *μ*M)	0.6 *μ*l	300 nM
Reverse primer (10 *μ*M)	0.6 *μ*l	300 nM
cDNA (2 *μ*g)	0.4 *μ*l	
Nuclease-Free Water	Up to 20 *μ*l	

**Table 5:** PCR amplification conditions.

Steps	Number of cycles	Temperature	Time	Detection
Predenaturation	1	95°C	10 min	Off
Denaturation	40	95°C	5 sec	Off
Annealing and extension		60°C	30 min	On

**Table 6:** Proportion of separating glue.

Name of reagents	Volume
DDW	4.825 mL
30% acrylamide	2.475 mL
1.5 M Tris HCl (pH 8.8)	2.5 mL
10% SDS	100 *μ*L
10% ammonium persulfate	100 *μ*L
TEMED	4 *μ*L

**Table 7:** Proportion of concentrated adhesive.

Name of reagents	Volume
DDW	2.225 mL
30% acrylamide	375 *μ*L
1.0 M Tris HCl (pH 6.8)	380 *μ*L
10% SDS	30 *μ*L
10% ammonium persulfate	30 *μ*L
TEMED	3 *μ*L

## Data Availability

The data used to support the findings of this study are available from the corresponding author upon request.

## References

[1] Luo A.-N., Qu Y.-Q., Dong R.-R. (2015). Progress in the treatment of breast cancer. *Progress in Modern Biomedicine*.

[2] Zhang J., Pei R., Pang Z. (2012). Prevalence and characterization of BRCA1andBRCA2 germline mutations in Chinese women with familial breast cancer. *Breast Cancer Research And Treatment*.

[3] Zhang J., Lisha A. I., Tingting L. V., Jiang X., Liu F. (2013). Asiatic acid, a triterpene, inhibits cell proliferation through regulating the expression of focal adhesion kinase in multiple myeloma cells. *Oncology Letters*.

[4] Jing Y., Wang G.-X., Zhou Q., Wei Y., Gong Z. (2018). Antiangiogenic effects of AA-PMe on HUVECs in vitro and zebrafish in vivo. *OncoTargets and Therapy*.

[5] Guo B.-J., Lin C.-Q. (2019). Antitumor effect of asiatidic acid and its mechanism. *Chinese Journal of Cancer Biotherapy*.

[6] Liao M.-J., Cheng H.-F. (2012). Development of PI3K/Akt/mTOR signal pathway inhibitor in breast cancer. *Chinese Journal of Cancer Prevention and Treatment*.

[7] Ye C. (2011). Summary and utilization of Actinidia valvata Dunn research. *China Pharmaceuticals*.

[8] Wan X.-Y., Zhang C., Lin C.-Q. (2004). The effect of Actinidia valvata Dunn injection on the function of liver cancer and its effect on the immune function. *Journal of Zhejiang Chinese Medical College*.

[9] Xu Y.-X., Xiang S.-B., Cheng X.-J. (2011). Anti-tumor constituents from the roots of *Actinidia valvata*. *Academic Journal of Second Military Medical University*.

[10] Tang L.-X., Yang G., Tan J.-J. (2009). Apoptosis of hepatic stellate cells induced by asiatic acid in rats. *Chinese Medicinal Herb*.

[11] Liu H.-Q., Xu Q.-T., Wang T.-L. (2014). Research progress of snow oxalic acid. *Wild Plant Resources in China*.

[12] Carvalho S., Schmitt F. (2010). Potential role of PI3K inhibitors in the treatment of breast cancer in the treatment of breast cance. *Future Oncology*.

[13] Osaki M., Oshimura M., Ito H. (2004). PI3K-Akt pathway: its functions and alterations in human cancer. *Apoptosis*.

[14] Edwards L.-A., Thiessen B., Dragowska W.-H., Daynard T., Bally M. B., Dedhar S. (2005). Inhibition of ILK in PTEN-mutant human glioblastomas inhibits PKB/Akt activation, induces apoptosis, and delays tumor growth. *Oncogene*.

[15] Song G., Ouyang G., Bao S. (2005). The activation of Akt/PKB signaling pathway and cell survival. *Journal of Cellular and Molecular Medicine*.

[16] Wang D.-S., Ching T.-T., Ppyrek J.-S., Chen C.-S. (2000). Biotinylated phosphati-dylinosipol 3, 4, 5-trisphosphate as affinity ligand. *Anal Analytical Biochemistry*.

[17] Tokunaga E., Oki E., Egashira A. (2008). Deregulation of the AKTpathway inhuman cancer. *Current Cancer Drug Targets*.

[18] Li Y., Corradetti M.-N., Inokiet K., Guanal K.-L. (2004). TSC2: filling the GAP in the mTOR signaling pathway. *Trends in Biochemical Sciences*.

[19] Sossey-Alaoui K., Li X., Ranalliet T.-A., Cowell J. K. (2005). WAVE 3-mediated cell migration and lamellipodia formation are regulated downstream of phosphatidylinositol3-kinase. *Journal of Biological Chemistry*.

[20] Wu Y.-N., Wang J.-H. (2014). The mechanism and research progress of wave3 in tumor invasion and metastasis. *Journal of Oncology*.

[21] Patil S. P., Maki S., Khedkar S. A., Rigby A. C., Chan C. (2010). Withanolide A and Asiatic Acid Modulate Multiple Targets Associated with Amyloid-*β* Precursor Protein Processing and Amyloid-*β* Protein Clearance. *Journal of Natural Products*.

[22] Lee Y. S., Jin D.-Q., Kwon E. J. (2002). Asiatic acid, a triterpene, induces apoptosis through intracellular Ca2+ release and enhanced expression of p53 in HepG2 human hepatoma cells. *Cancer Letters*.

[23] Wu T., Geng J., Guo W. (2017). Asiatic acid inhibits lung cancer cell growth in vitro and in vivo by destroying mitochondria. *Acta Pharmaceutica Sinica B*.

[24] Ren L., Cao Q.-X., Zhai F.-R. (2016). Asiatic Acid Exerts Anticancer Potential in Human Ovarian Cancer Cells via Suppression of PI3K/Akt/mTOR Signalling. *Pharmaceutical Biology*.

[25] Thakor F. K., Wan K.-W., Welsby P. J., Welsby G. (2017). Pharmacological effects of asiatic acid in glioblastoma cells under hypoxia. *Molecular and Cellular Biochemistry*.

[26] Wang Z.-H. (2014). Anti-glycative effects of asiatic acid in human keratinocyte cells. *BioMedicine*.

[27] Lee Y. S., Jin D. Q., Beak S. M. (2003). Inhibition of ultraviolet-A-modulated signaling pathways by asiatic acid and ursolic acid in HaCaT human keratinocytes. *European Journal of Pharmacology*.

[28] Lu Y., Kan H., Wang Y. (2018). Asiatic acid ameliorates hepatic ischemia/reperfusion injury in rats via mitochondria-targeted protective mechanism. *Toxicology and Applied Pharmacology*.

[29] Wei L., Chen Q., Guo A., Fan J., Wang R., Zhang H. (2018). Asiatic acid attenuates CCl 4 -induced liver fibrosis in rats by regulating the PI3K/AKT/mTOR and Bcl-2/Bax signaling pathways. *International Immunopharmacology*.

[30] Zhang X., Wu J., Dou Y. (2012). Asiatic acid protects primary neurons against C2-ceramide-induced apoptosis. *European Journal of Pharmacology*.

[31] Lee K. Y., Bae O.-N., Weinstock S., Kassab M., Majid A. (2014). Neuroprotective effect of asiatic acid in rat model of focal embolic stroke. *Biological and Pharmaceutical Bulletin*.

[32] Raval N., Barai P., Acharya N., Acharya S. (2018). Fabrication of peptide-linked albumin nanoconstructs for receptor-mediated delivery of asiatic acid to the brain as a preventive measure in cognitive impairment: optimization, in-vitro and in-vivo evaluation. *Artificial Cells, Nanomedicine, and Biotechnology*.

